# Interaction of βA3-Crystallin with Deamidated Mutants of αA- and αB-Crystallins

**DOI:** 10.1371/journal.pone.0144621

**Published:** 2015-12-11

**Authors:** Ekta Tiwary, Shylaja Hegde, Sangeetha Purushotham, Champion Deivanayagam, Om Srivastava

**Affiliations:** 1 Department of Vision Sciences, School of Optometry, University of Alabama at Birmingham, Birmingham, Alabama, 35294, United States of America; 2 Department of Vision Sciences/Centre for Structural Biology, University of Alabama at Birmingham, Birmingham, Alabama, 35294, United States of America; Tsinghua University, CHINA

## Abstract

Interaction among crystallins is required for the maintenance of lens transparency. Deamidation is one of the most common post-translational modifications in crystallins, which results in incorrect interaction and leads to aggregate formation. Various studies have established interaction among the α- and β-crystallins. Here, we investigated the effects of the deamidation of αA- and αB-crystallins on their interaction with βA3-crystallin using surface plasmon resonance (SPR) and fluorescence lifetime imaging microscopy-fluorescence resonance energy transfer (FLIM-FRET) methods. SPR analysis confirmed adherence of WT αA- and WT αB-crystallins and their deamidated mutants with βA3-crystallin. The deamidated mutants of αA–crystallin (αA N101D and αA N123D) displayed lower adherence propensity for βA3-crystallin relative to the binding affinity shown by WT αA-crystallin. Among αB-crystallin mutants, αB N78D displayed higher adherence propensity whereas αB N146D mutant showed slightly lower binding affinity for βA3-crystallin relative to that shown by WT αB-crystallin. Under the in vivo condition (FLIM-FRET), both αA-deamidated mutants (αA N101D and αA N123D) exhibited strong interaction with βA3-crystallin (32±4% and 36±4% FRET efficiencies, respectively) compared to WT αA-crystallin (18±4%). Similarly, the αB N78D and αB N146D mutants showed strong interaction (36±4% and 22±4% FRET efficiencies, respectively) with βA3-crystallin compared to 18±4% FRET efficiency of WT αB-crystallin. Further, FLIM-FRET analysis of the C-terminal domain (CTE), N-terminal domain (NTD), and core domain (CD) of αA- and αB-crystallins with βA3-crystallin suggested that interaction sites most likely reside in the αA CTE and αB NTD regions, respectively, as these domains showed the highest FRET efficiencies. Overall, results suggest that similar to WT αA- and WTαB-crystallins, the deamidated mutants showed strong interactionfor βA3-crystallin. Variable in vitro and in vivo interactions are most likely due to the mutant’s large size oligomers, reduced hydrophobicity, and altered structures. Together, the results suggest that deamidation of α-crystallin may facilitate greater interaction and the formation of large oligomers with other crystallins, and this may contribute to the cataractogenic mechanism.

## Introduction

Crystallins (α- and β-γ- superfamily) are the major structural proteins of the vertebrate lens, and are responsible for maintenance of lens transparency [1]. Among them, α-crystallin forms a large oligomer (up to 800 kDa), and composed of αA- and αB- subunits (20 kDa each) [2,3]. αA- and αB crystallins share 60% sequence homology, and are small heat shock proteins with chaperone activity. The β-γ superfamily is comprised of structural proteins, constituted by acidic (βA3/βA1, βA2 and βA4) and basic (βB1, βB2 and βB3) β-crystallins and γ-crystallins (γA, γB, γC, γD, γE and γF) [1], and they share conserved homologous sequences. β-crystallins form heterogeneous oligomers while the γ-crystallins are monomers. The expression of these crystallins is developmentally and spatially regulated, and their short-range order interaction is critical for transparency and refractive power of the lens [4,5].

During aging and cataract development, various mutations and age-related post-translational modifications (PTMs) occur in the crystallins. Examples of such PTMs include photooxidation, deamidation, disulfide bond formation, and cleavage [6,7]. The PTMs result in incorrect interactions, oligomerization, aggregation, cross-linking, and insolubilization of crystallins, which may lead to the development of lens opacity [6–11]. Misfolding, deletion, and premature termination of crystallins have been demonstrated to be associated with the human inherited autosomal, dominant, congenital zonular, or nuclear sutural cataracts [12–14]. Some mutations such as splice site-, point-, or nonsense mutations have also been reported in various autosomal dominant-, congenital zonular-, and nuclear sutural cataracts in human and mouse models [15,16]. PTMs such as truncations of the crystallins can lead to altered solubility, oligomerization, and supra-molecular assembly, which are believed to be causative factors for cataract development. For example, truncation of 51 residues from the C-terminal region of the CRYBB2 gene mutant (Q155) have been shown to cause cerulean cataract [17]. Studies have shown that altered crystallin structures could lead to abnormal interactions with other crystallins and to cataract development.

Deamidation of crystallins is one of the major PTM’s that occurs during aging and cataract development. Deamidation alters the tertiary structure of crystallins and affects their structural and functional properties [18,19]. While Gln and Asn are susceptible to deamidation, Asn is three-times more prone to deamidation than Gln [20]. Several studies have shown in vivo deamidation of α-, β-, and γ-crystallins [21–26]. Deamidation of αA-crystallin occurs at Gln-6, Gln-50, Asn-101, and Asn-123 residues [18,22,25]. The deamidation at Asn-101 and Asn-123 residues in αA-crystallin altered its structure, formed larger oligomers, and reduced chaperone activity [27]. Similarly, αB-crystallin with deamidation at Asn-146 showed reduced chaperone activity, altered conformation, and increased oligomer sizes relative to the wild type protein [28]. In contrast to the deamidation at Asn-146, deamidation of the αB-crystallin at Asn-78 showed relatively moderate changes in structural and functional properties [29]. In addition to Asn residue, deamidation of Gln was also reported in αB-crystallin [22]. Similarly, deamidations of βA3-, βB1-, γS- and γD-crystallins have also been reported [23,26,30–32]. Together, these studies suggest that deamidation of crystallins alters their stability and forms high molecular weight aggregates.

α-crystallin has 3 distinct domains, i.e. N-terminal domain (NTD), core domain (CD), and C-terminal extension (CTE). The N-terminal domain (NTD) and core domain (CD) of α-crystallin have been reported as substrate binding sites for chaperone activity [33–38]. The CTE of α-crystallins has been reported to be involved in the recognition and selection of unfolded protein substrates; the CTE of αB-crystallin has also been identified as a substrate binding site [39]. In addition to chaperone activity, α-crystallin has also been reported to inhibit trypsin, elastase [40], caspase-3 [41,42], and an endogenous lens proteinase [43]. The C-terminal extension has been assumed to be an inhibitor of trypsin [44].

It is now well established that PTMs of crystallins including deamidation affect their interaction and result in the loss of lens transparency [5]. Therefore, it is important to characterize the effect of deamidation on crystallin-crystallin interactions. We previously demonstrated that βA3-crystallin isolated from α-crystallins fraction exhibited protease activity after it’s dissociate from α-crystallin [45]. This result implied that the α-crystallin is most likely acting as an inhibitor of the βA3-crystallin’s protease activity. Furthermore, we also demonstrated the interaction of βA3-crystallin with αA- and αB-crystallins and identified the interaction sites of βA3-crystallin [46]. To further extend our previous studies, here we have analyzed in vitro interaction of WT and deamidated αA- and αB-crystallins with βA3-crystallin by SPR method, and also in vivo interaction by FLIM-FRET method in HeLa cells. SPR analysis is selected because it identifies even the weak interactions and also quantifies molar association and dissociation rates. Similarly, the FLIM-FRET method could identify interactions among crystallins in vivo under the physiological condition in cells. As mentioned above, NTD, CD and CTE of α-crystallins are known to be involved in substrate binding, recognition and selection of unfolded substrates therefore, it is valuable to identify the interacting regions of α-crystallins with βA3-crystallin. Using the FLIM-FRET method, we identified αA- and αB-crystallins domains (NTD, CD and CTE regions) that interact with the βA3-crystallin.

## Materials and Methods

### Materials

The mammalian expression vectors pAm Cyan1-N1 and pZS Yellow1-N1, the Hi-Fi PCR mix, and the infusion enzyme were obtained from Clontech Laboratories (Mountain View, CA) to generate fluorescently-tagged fusion proteins. Cell culture reagents were obtained from Invitrogen (Carlsbad, CA), and a CM-5 chip was purchased from GE-Biosciences (Arlington Heights, IL). HeLa cells were a kind gift from Dr. Vincenzo Guarcello (Department of Vision Sciences, University of Alabama at Birmingham, Birmingham, AL, USA), and Dr. Scott W. Blume (Department of Medical Hematology and Oncology, University of Alabama at Birmingham, Birmingham, Al, USA). Primers used in the study were synthesized by Sigma-Aldrich (St. Louis, MO). Profinity IMAC Ni-charged resins, DNA, and protein markers were from Bio-Rad (Hercules, CA), and the site-directed mutagenesis kit was from Stratagene (Agilent Technologies Inc, CA). Restriction enzymes and T4 DNA ligase were obtained from New England Biolabs Inc (Ipswich, MA) and Thermo Fisher Scientific (Atlanta, GA). Unless indicated otherwise, all other chemicals used were purchased from Thermo Fisher Scientific (Atlanta, GA).

### Construction of Recombinant Proteins

#### Ligation method

Genes were cloned in pET 28b and pZS Yellow1-N1 vectors using a ligation method as described below. Desired genes were amplified by polymerase chain reaction (PCR) with appropriate primers in a 25 μl reaction mixture containing 2.5 μl of Taq buffer (10X), 20 pmoles of forward and reverse primers, 25 ng of template DNA, 0.2 μl of DNTPs (10 mM), and 0.5 μl of Taq DNA polymerase. The following PCR conditions were used: pre-denaturation at 95°C for 5 min, 30 cycles of denaturation at 95°C for 30 sec, annealing at 55–60°C for 30 sec (depending upon the Tm of the primers), and extension at 72°C for 1 min with a final extension at 72°C for 5 min. PCR products were purified by a gel-elution method using a gel extraction kit (Qiagen, Hilden, Germany) and treated with *Nhe* I and *Xho* I for 1 h at 37°C. Simultaneously, the vector was also linearized with *Nhe* I and *Xho* I, and all the reactions were inactivated. The restriction enzyme-treated DNA was gel-purified. Further, a 3:1 insert vector ratio was used for the ligation with T4 DNA ligase, and the ligation mixture was incubated at room temperature for 1 h. Next, the ligation mixture (2–5 μl) was transformed to 20 μl *E*. *coli* XL-10. All constructs were confirmed by DNA sequencing at the Genomics Core Laboratory of the University of Alabama at Birmingham.

#### Infusion method

All the constructs in mammalian expression vectors, i.e. pAm Cyan1-N1 and pZS Yellow1-N1, were generated by an infusion method. Vectors were linearized with *Nhe* I and *Xho* I and purified by the gel-elution method. The desired genes were PCR amplified with the appropriate primers in a 25 μl reaction mixture containing 12.5 μl of Hi Fi PCR mix, 10 pmoles of forward and reverse primers, and 5–20 ng of template DNA. Amplifications were performed under the following PCR conditions: denaturation at 98°C for 10 sec, annealing at 55°C for 15 sec, and extension at 72°C for 5 sec for 30 cycles. Amplicons were purified by the gel-elution method. Further, 10 μl reaction mixtures containing 100 ng of linearized vector, 50 ng of insert, and 2 μl of 5X Infusion HD enzyme premix were incubated at 50°C for 15 min, and placed on ice. Transformations were performed in 20 μl stellar competent cells (Clontech, Mountain View, CA) with a 2 μl infusion mixture using a standard transformation method. Recombinant bacteria were selected on a kanamycin agar plates, and plasmids were isolated from 4 random colonies. Constructs were verified by DNA sequencing at the Genomics Core Laboratory of the University of Alabama at Birmingham.

#### Site-directed mutagenesis

Deamidation of Asn to Asp residue in αA- and αB-crystallins was performed using a site-directed mutagenesis kit (Quickchange; Stratagene, La Jolla, CA) by following the manufacturer’s instructions. The pET 28b constructs and pZS Yellow1-N1 constructs of αA- and αB-crystallins were used as templates with the appropriate primers. The desired mutations were confirmed by DNA sequencing at the DNA core facility as described above.

### In Vitro Interaction of βA3-Crystallin with αA- and αB-Crystallins and Their Deamidated Mutants

#### Cloning, expression and purification of proteins

Recombinant His-tagged WT βA3-, WTαA-, and WTαB-crystallins were generated in a pET 28b vector using the ligation method as described above. The deamidated mutants of αA- and αB-crystallins (αA N101D, αA N123D, αB N78D and αB N146D) were generated by site-directed mutagenesis using appropriate primers (Table 1). Proteins were expressed in *E*. *coli* pLys S BL-21 cells at 37°C using the IPTG method, and the expressed proteins were released by cell lysis. All the proteins were purified using the Ni^2+^-affinity column chromatographic method as described earlier [47]. Purified proteins were dialyzed against the phosphate buffer (50 mM sodium phosphates, pH 7.8, 150 mM NaCl), and concentrated using 10 kDa cut off centricon tubes (EMD-Millipore, Billerica, MA). Freshly purified proteins with >80–90% purity were confirmed by SDS-PAGE. The minor bands were identified as oligomers by western blot analysis, and were used for the SPR study.

**Table 1:** List of primers used for the generation of recombinant His-tagged crystallins.

Primers	Forward	Reverse
**βA3**	5’CTAGCTAGCATGGAGACCCAGCTGAG3’	5’CCGCTCGAGCGGCTACTGTGGATTGGATTCGGCGA3’
**αA**	5’CTAGCTAGCATGGACGTGACCATCCAGC3’	5’CCGCTCGAGCGGTTAGGACGAGGGAGCCGA3’
**αB**	5’CTAGCTAGCATGGACATCGCCATCCAC3’	5’CCGCTCGAGCGGCTATTTCTTGGGGGCTGC3’
**αA N101D**	5’ATCCACGGAAAGCACGACGAGCGCCAGGACGACC3’	5’GGTCGTCCTGGCGCTCGTCGTGCTTTCCGTGGAT3’
**αA N123D**	5’GCTACCGCCTGCCGTCCGACGTGGACCAGTCGGCC3’	5’GGCCGACTGGTCCACGTCGGACGGCAGGCGGTAGC 3’
**αB N78D**	5’GACAGGTTCTCTGTCGACCTGGATGTCAAGCAC3’	5’GTGCTTCACATCCAGGTCGACAGAGAACCTGTC3’
**αB N146D**	5’GGGGTCCTCACTGTGGACGGACCAAGGAAACAGG3’	5’CCTGTTTCCTTGGTCCGTCCACAGTGAGGACCCC3’

#### Interaction between βA3- and αA-/αB-crystallins and their deamidated mutants by SPR

Interactions between WT βA3- and WTαA-/WT αB-crystallins were analyzed by SPR using the BIACORE 2000, (GE Healthcare Bio-Sciences, Piscataway, NJ). The CM5 sensor chip (GE Healthcare) was activated using 1:1 ratio of 0.4 M 1-ethyl-3-(3-dimethylamino-propyl)-carbodiimide hydrochloride (EDC) and N-hydroxysuccinimide (NHS) at a flow rate of 20 μl/min. βA3-crystallin (200 μg/ml) was diluted to 50 μg/ml with 10 mM sodium acetate buffer (pH 5.0),and immobilized on the sensor chip to ~2500 RU, where 1 RU is estimated to be approximately equal to 1 pico gram of βA3-crystallin. The residual unreacted NHS was inactivated using 1 Methanolamide. A control flow cell was also activated with EDC/NHS (1:1) mixture and blockedwith 1 M ethanolamide.

Individual analytes αA- (WT, αA N101D, αA N123D) as well as αB- (WT, αB N78D, αB N146D) were flowed over the immobilized βA3-crystallin surface at varying concentrations (5 μM-25 μM) at a flow rate of 20 μL/min. The interactions between the various components were recorded and after each binding cycle, the chip surface was regenerated with 1 M NaCl. All experiments were carried out in duplicates at room temperature with 50 mM sodium phosphate buffer, pH 7.8 containing 150 mM NaCl as the running buffer. The concentration of the proteins was a limiting factor in these experiments, as they tended to aggregate readily even at very low concentrations. Given the limitations, these experiments were carried out and the curve fitting were analysed with BIA evaluation software 8.1 [48]. The observed variations in RU for any given concentration indicated that there could be additional non-specific adhesion events that occur during and after the initial interaction. While many different binding models were utilized to fit the curves, we have chosen to report here the simplistic 1:1 Langmuir kinetics, with the cautionary note that one cannot overly emphasize the fitted kinetic parameters due to the complex nature of the interactions that exist between βA3- and WTαA-/WTαB-crystallins and their deamidated mutants. Currently there exists no curve fitting protocol to delineate and determine kinetics of specific and non-specific interactions. Therefore instead of reporting fitted affinities, we have analysed the adherence propensities. In this analysis, the average RU values after injection of the analyte between 260–270 seconds (where the peak plateaus towards saturation for each concentration), were analyzed and plotted for each concentration. The utilization of the same immobilized chip surface provided the basis for this type of analysis.

### Analysis of Interaction of βA3-Crystallin with αA-, αB-Crystallins and Their Deamidated and Domain Mutants by FLIM-FRET Method

#### Cloning of WT βA3-, WT αA-, WT αB-crystallins and mutants in mammalian expression vectors

The pAm Cyan1-N1 (designated as CFP) and pZS Yellow1-N1 (designated as YFP) mammalian expression vectors were used for protein expression in HeLa cells. Clones of YFP αA CD (64–142 amino acids) and YFP αB CD (65–146 amino acids) were generated by the ligation method as described above. CFP WT βA3-, YFP WT αA-, YFP WT αB-, YFP αA NTD- (1–63 amino acids), YFP αA CTE- (143–173 amino acids), YFP αB NTD- (1–64 amino acids), and YFP αB CTE- (147–175 amino acids) crystallins were generated using appropriate primers (Table 2), and an infusion method as described above. Deamidated mutants, i.e. αA N101D, αA N123D, αB N78D and αB N146D, were generated by the site-directed mutagenesis method using appropriate primers (Table 1). For a positive control, YFP was cloned in a CFP vector at the *Nhe* I/*Xho* I restriction site using an infusion method with YFP forward and reverse primers at a 12 amino acid distance from the CFP. Unlinked CFP- and YFP- vectors were used as negative control. The CFP-tagged protein (CFP βA3-crystallin) was used as a donor, and the YFP-tagged proteins were used as acceptors in the FLIM-FRET analysis.

**Table 2:** List of primers used for generation of recombinant crystallins in mammalian expression vectors.

Primers	Forward	Reverse
**βA3- CFP**	5’GAACCGTCAGATCCGCTAGCGATGGAGACCCAGGCT3’	5’ACGAAGCTTGAGCTCGAGCTGTTGGATTCGGCG3’
**αA NTD-YFP**	5’GAACCGTCAGATCCGCTAGCGATGGACGTGACCATC3’	5’ACGAAGCTTGAGCTCGAGCTCAGAGATGCCGGA3’
**αA CD-YFP**	5’CGCTAGCGATGGTTCGATCCGACCGG3’	5’CTCGAGACAGAAGGTCAGCATGCCATC3’
**αA CTE-YFP**	5’GAACCGTCAGATCCGCTAGCGATGGGCCCCAAGATCCAG3’	5’CGAAGCCTTGAGCTCGAGGGACGAGGGAGCCGAGGTGGG3’
**αB NTD-YFP**	5’GAACCGTCAGATCCGCTAGCGATGGACATCGCCATC3’	5’ACGAAGCTTGAGCTCGAGTCCAGTGTCAAACCA3’
**αB CD-YFP**	5’CGCTAGCGATGCTCTCAGAGATGCGC3’	5’CTCGAGATTCACAGTGAGGACCCC3’
**αB CTE-YFP**	5’GAACCGTCAGATCCGCTAGCGATGGGACCAAGGAAACAG3’	5’ACGAAGCTTGAGCTCGAGTTTCTTGGGGGCTGC3’

#### Tissue culture and transfection

HeLa cells were grown in a modified Eagle’s medium with high glutamate (MEM Glutamax, Invitrogen, Carlsbad, CA) supplemented with 10% fetal bovine serum (FBS) at 37°C with 5% CO_2_. For transfection, 1X10^5^ cells were seeded in 35 mm μ-dishes (35μ-dish, Ibidi, Germany) in 800 μl medium and were grown for 18 h or until they reached up to 80% confluency. For transfection, 3 μl lipofectamine 2000 (Invitrogen, Carlsbad, CA), 2 μg of donor DNA (CFP βA3-crystallin), and 2 μg of acceptor (YFP constructs) were mixed with 200 μl of Opti-MEM medium (Phenol red free DMEM) and incubated at room temperature for 25 min. After 25 min, the volume of the lipofectamine–DNA mixture was maintained up to 800 μl. Cells were washed with phosphate buffer saline (PBS), and 800 μl transfection mixtures were added to the cells and incubated at 37°C with 5% CO_2_ for 5–6 h. Next, the transfection mixture was supplemented with 10% FBS and incubated for 18–20 h at 37°C with 5% CO_2_. Expressions of the CFP-tagged and YFP-tagged proteins were examined using a Zeiss Axioplan2 fluorescence microscope at the Vision Sciences Research Centre Ocular Phenotyping and Molecular Analysis core facility at the University of Alabama at Birmingham.

#### Western blot analysis

After 24 h of transfection, cells were lysed with 100 μl radio immunoprecipitation assay (RIPA) buffer (Fisher-Thermo Scientific) containing a cocktail of protease inhibitors (Roche). Twenty μl of lysates were then loaded onto 15% SDS-PAGE gels, and after electrophoresis, electro-blotted to a PVDF membrane by the Trans-Blot Turbo transfer system (Bio-Rad). Next, the blots were blocked with 3% bovine serum albumin prepared in PBST (Phosphate buffer saline supplemented with 1% v/v Tween 20) for 1 h, and subsequently incubated with a primary polyclonal anti-βA3-crystallin antibody (1:500 dilution, Santa Cruz Biotechnology, Dallas, TX), or monoclonal anti-αA- or anti αB-antibodies (1:1000 dilution, Abcam, Cambridge, MA) for 1 h at room temperature. Blots were washed three times with PBST and incubated in the dark with an appropriate secondary antibody (IR-dye conjugated, Li-Cor, Lincoln, NE) for 1 h at room temperature. Signals were detected by exposing the blots to 700 and 800 channels using a Li-Cor Oddessy instrument (Lincoln, NE). β-actin was used as a loading control, and the membrane was probed with a rabbit polyclonal antibody against β-actin (1:1000 dilution, Cell Signaling Technology, Danvers, MA) using the protocol described above.

#### FLIM-FRET imaging

Live HeLa cells plated onto μ-dishes were subjected to confocal FLIM imaging using a Becker and Hick GmbH pulsed diode 405 nm with a simple Tau Time correlated single photon counting module attached to a Zeiss LSM 710 confocal microscope at the High Resolution Imaging core facility of the University of Alabama at Birmingham. Confocal imaging was performed with the Zeiss microscope to detect the localization of CFP-tagged and YFP-tagged proteins. FRET was performed by exciting the CFP-tagged donor (CFP βA3-crystallin throughout the FRET) with a 405 ps pulsed diode laser with an 80 MHz repetition rate. Lifetime images were obtained by selecting a region of interest containing both CFP-tagged- and YFP-tagged proteins, and images were analyzed by SPC Image software to obtain the life-time of the CFP. A double exponential decay analysis was performed for a single measurement point. For each experiment, 5–6 individual cells were imaged from 3 individual samples, analyzed for mean CFP-life-time and average life-time. FRET efficiency was calculated by E = 1-T_FRET_/T_CFP_, where T_FRET_ and T_CFP_ were the CFP life-times obtained from the cells expressing both (CFP and YFP) and CFP alone, respectively.

## Results

### Purification of βA3-, WT αA- and WT αB-Crystallins and Their Mutants

**Fig 1:**
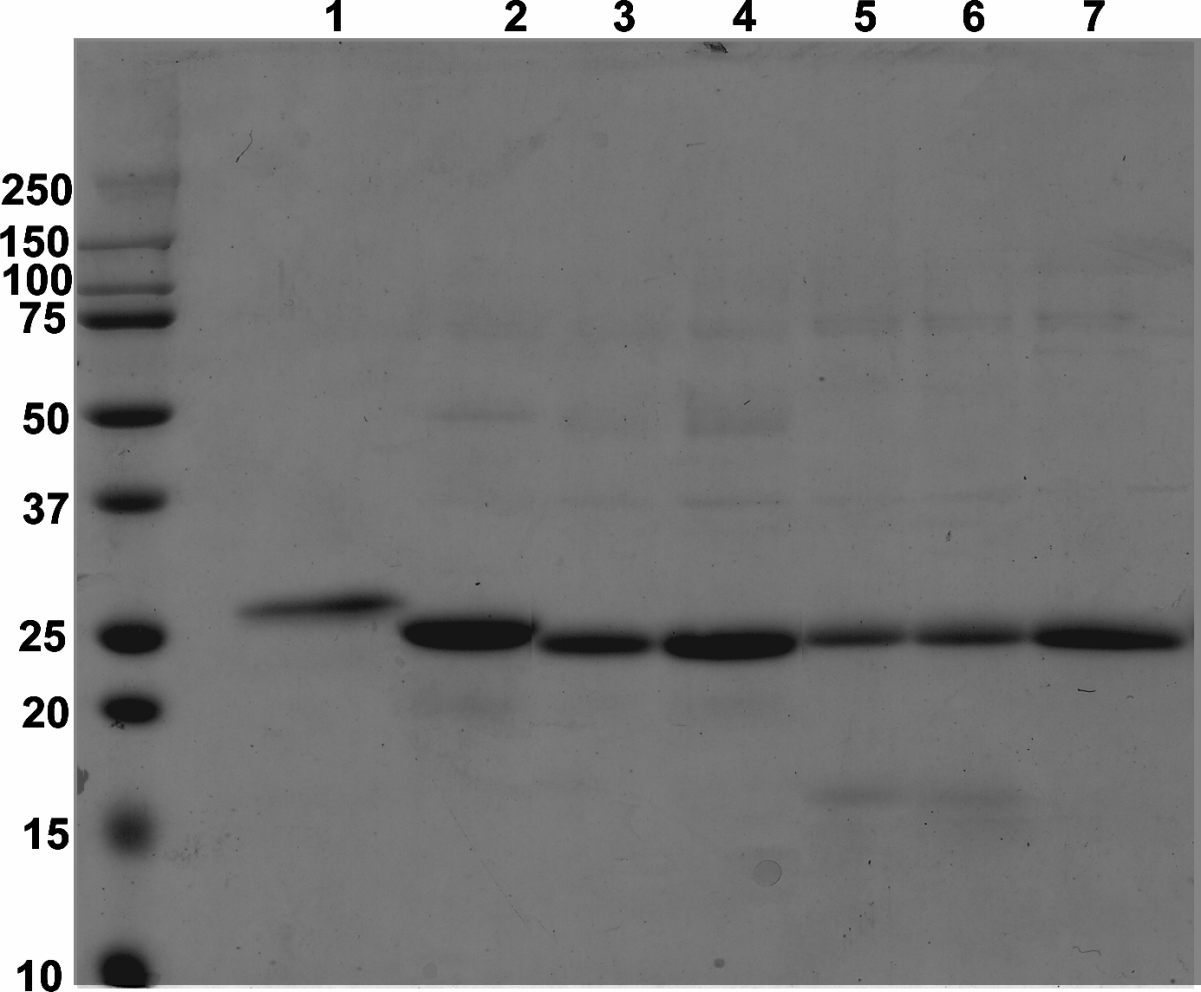
SDS-PAGE profile of purified βA3-, αA- and αB-crystallins and their deamidated mutants. Lane 1: βA3-crystallin, lane 2: WT αA-crystallin, lane 3: αA N101D, lane 4: αA N123D, lane 5: WT αB-crystallin, lane 6: αB N78D, lane 7: αB N146D.

Recombinant proteins were purified using the Ni-affinity chromatographic method, and the purity of each protein was monitored by SDS-PAGE analysis (Fig 1). Each of the protein preparation with >80–90% purity was recovered and concentrated. α-crystallins formed oligomers which appeared as minor bands and was confirmed by western blot (data not shown). These >80–90% pure proteins were used for the SPR analysis.

### SPR Analysis of βA3-Crystallin Interaction with WT αA- and WT αB-Crystallins and Their Deamidated Mutants

#### Effect of deamidation at Asn 101 and Asn123 position of WT αA- on the binding of βA3-crystallin

**Fig 2:**
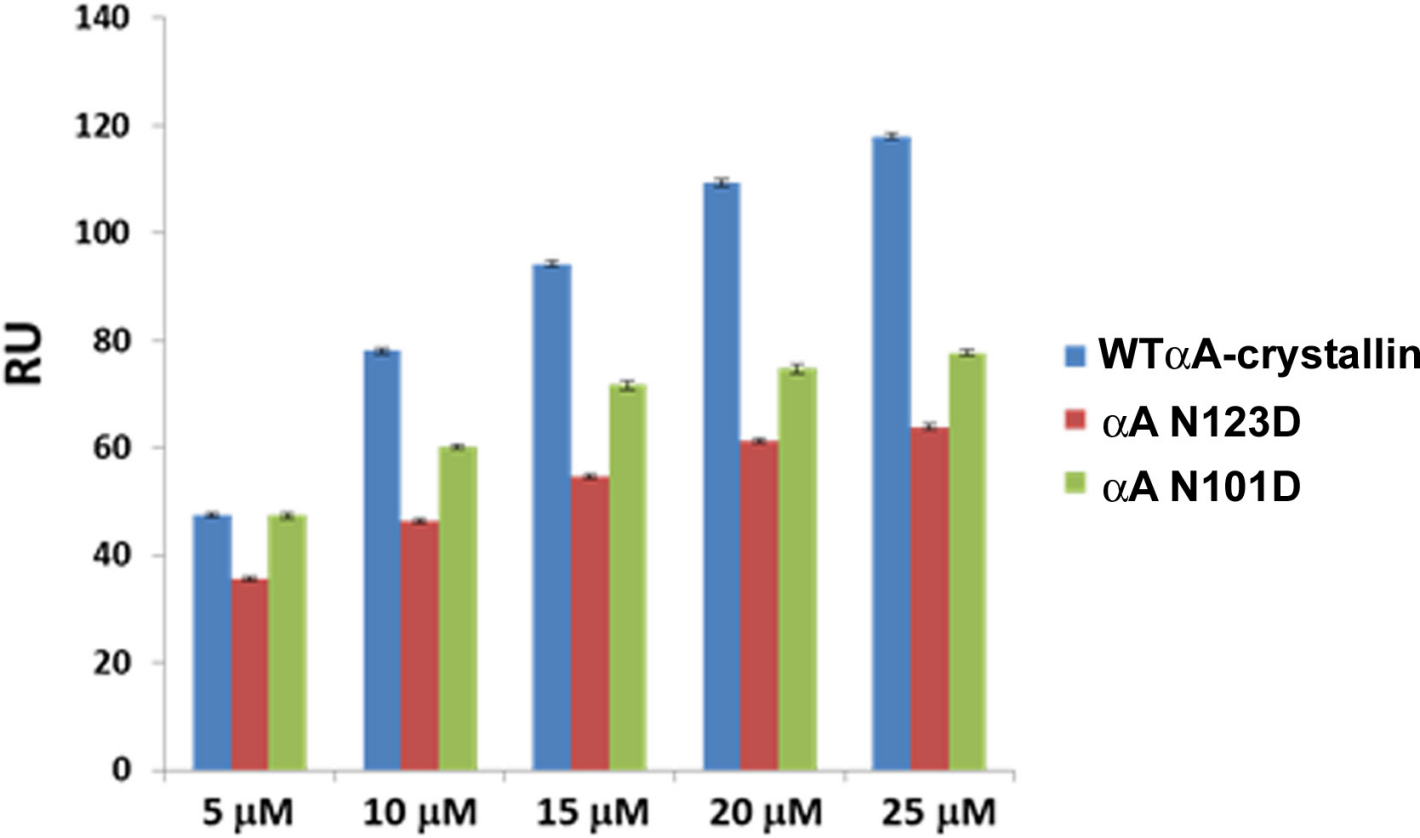
SPR assay of the binding of WT αA-, αA N101D and αA N123D crystallins with βA3- crystallin. Binding responses (average RU obtained between 260–270 sec of association) of at 5, 10, 15, 20 and 25 μM of analytes (WTαA-crystallin, αA N101D and αA N123D) with βA3-crystallin.

The interaction of WT αA- and its deamidated mutants with the βA3-crystallin was studied using 5 μM to 25 μM of analytes. S1 Fig display sensograms for the observed interactions of WT αA-, αA N101D and αA N123D with βA3-crystallin, respectively. The fitted curves for WT αA-, αA N101D and αA N123D indicated very high affinity interactions with βA3-crystallin (K_D_ values of 1.63X10^-9^ M, 5.20X10^-9^ M and 5.09X10^-9^ M, respectively). As explained earlier in the Methods section, utilization of various binding models did not yield better fitting to the observed results. The currently existing fitting protocols were ineffective in delineating the complex interactions (specific/non-specific) between the molecules, and we therefore report binding propensities based on average RU’s observed of the analyte between 260–270 sec where the RU’s plateau (or saturate) for any given concentration (Fig 2). As shown in Fig 2, WT αA-adherence was relatively higher at each concentration compared to αA N101D and αA-N123D mutants. The adherence propensities of WT αA- and it’s deamidated mutants are ranked as WT αA> αA N101D> αAN123D. This suggested that deamidation of Asn101 and Asn123 to Asp decreased the binding affinity of WT αA-crystallin for βA3-crystallin.

#### Effects of deamidation of αB-crystallins on the binding of βA3-crystallin

**Fig 3:**
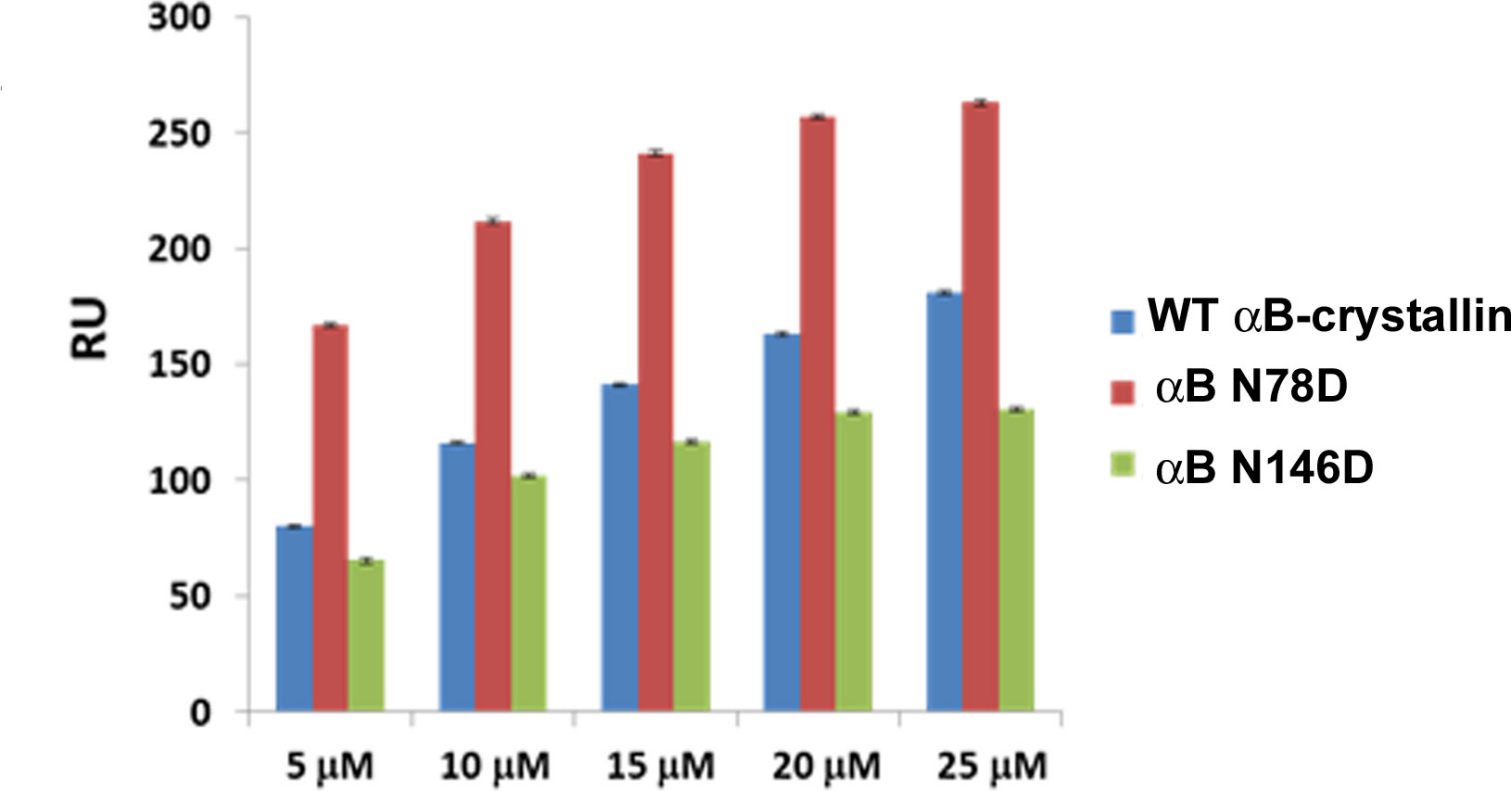
SPR assay of the binding of WT αB-, αB N78D and αB N146D crystallin with βA3-crystallin. Binding responses (average RU obtained between 260–270 sec of association) of at 5, 10, 15, 20 and 25 μM of analytes (WTαB-crystallin, αB N78D and αB N146D) with βA3-crystallin.

Similar to αA-crystallin, very high affinities were derived for the interactions between WT αB-crystallin (5.75 × 10^−9^ M) and it’s deamidated mutants αB N78D (2.46 × 10^−9^ M)and αB N146D (5.05 ×10^−9^ M) with immobilized βA3-crystallin from fitting the observed sensorgrams (S2 Fig). αB N78D showed highest propensity to adhere with βA3-crystallin at each concentration (Fig 3). The binding of αB-crystallin and it’s deamidated mutants with βA3-crystallin are ranked in the order αB N78D>WT αB> αB N146D. Thus, in case of αB-crystallin, deamidation at N78D position increasingly adheres with βA3-crystallin.

In summary, WTαA-, WTαB-crystallin and their deamidated mutants diplayed higher affinity interactions with βA3-crystallin. However, deamidated mutants of αA-crystallin exhibited comparatively lower binding propensities for βA3-crystallin than WT αA-crystallin, whereas the αB N78D mutant had a higher binding affinity relative to the WT αB and the αB N146D mutant.

### In Vivo Interaction of βA3-Crystallin with αA- and αB-Crystallins and Its Mutants by FLIM-FRET Method

The in-vivo interaction of WTβA3-crystallin with WTαA- and WTαB-crystallins was determined by measuring the life-time of a donor by exciting the fluorescent dye of the donor (CFP βA3-crystallin) in the presence and absence of an acceptor (YFP WT αA- or WT αB-crystallins or their mutants). When the acceptor and donor are at <10 nm distance and they interact, then the energy of the donor is transferred to the acceptor, and a decrease in the life-time of the donor could be observed. An YFP-CFP fusion protein separated by a 12-amino acid linker was used as a positive control. CFP co expressed with YFP, and CFP βA3-crystallin co-expressed with YFP were used as negative controls. Donor and acceptor proteins co-expressed in cytoplasm were selected as the region of interest for FRET analysis.

#### Expression of proteins in HeLa cells

**Fig 4:**
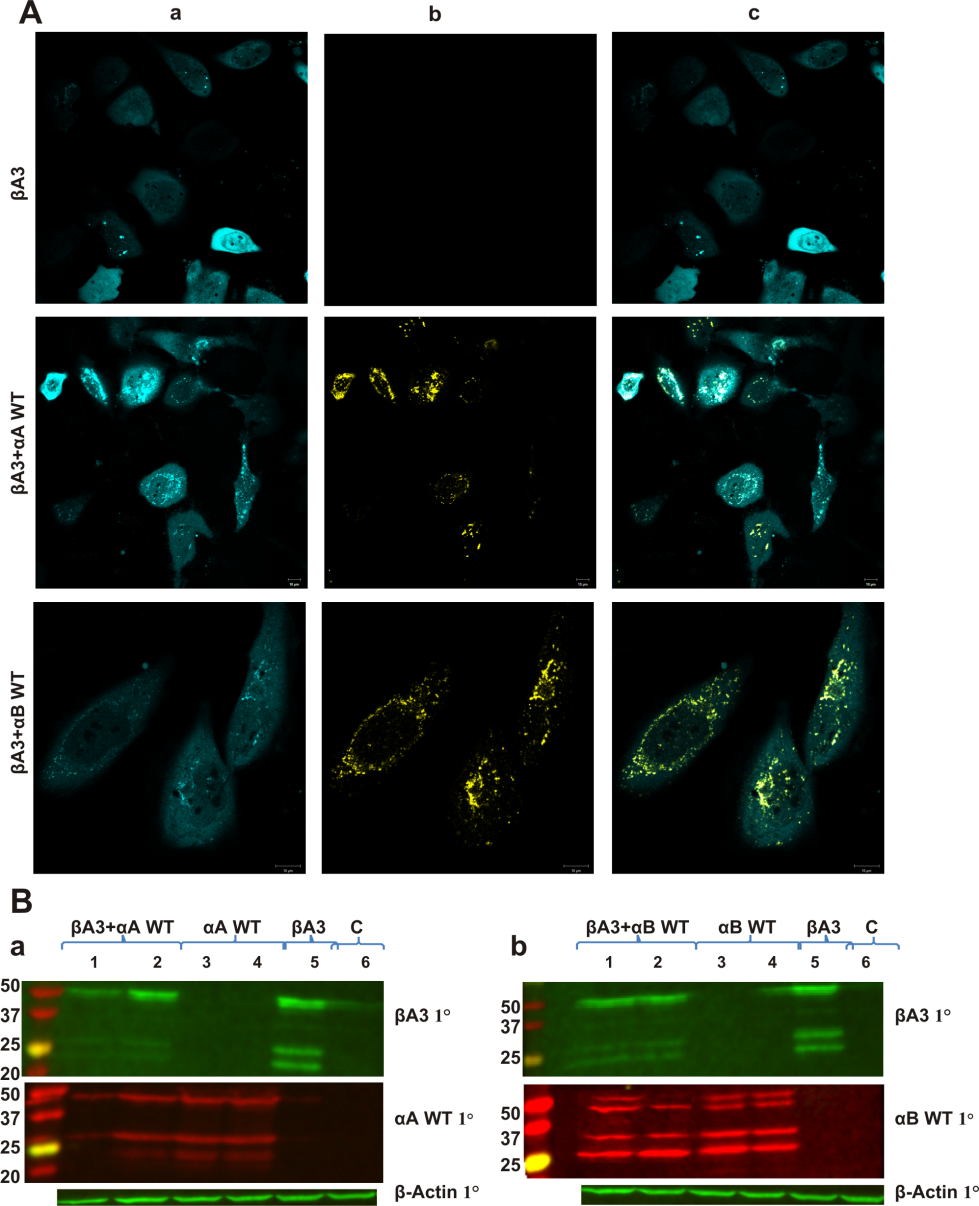
Confocal images of expression of αA- and WT αB- crystallin with βA3- crystallin in HeLa cells. Confocal microscopic images of transfected HeLa cells with: **A.** CFP βA3-crystallin alone and co-transfected with CFP βA3-crystallin-YFP WT αA-crystallin; and CFP βA3-crystallin-YFP WT αB-crystallin. Note the co-expression of CFP- and YFP-tagged crystallins. Panel a: CFP channel image of cells co-transfected with pairs of CFP- and YFP-fusion crystallins Panel b: YFP channel image of cells co-transfected with pairs of CFP- and YFP- fusion crystallins Panel c: Merged images for CFP- and YFP-channels of cells co-transfected with pairs of CFP- and YFP-fusion crystallins. Note that in case of CFP βA3-crystallin alone, no-bleed through expression of YFP was observed. **B.** Western blot analysis of the expression of **a.** CFP βA3-crystallin and YFP WT αA-crystallin Lanes 1 and 2: αA+ βA3, lanes 3 and 4: αA-, lane 5: βA3, Lane 6:Control. **b.** CFP βA3-crystallin and YFP WT αB-crystallin Lanes 1 and 2: αB+ βA3, lanes 3 and 4: αB-, lane 5: βA3, lane 6:Control. using βA3-1° antibody (green) and αA/ αB 1° antibody (red). β-actin was used as a loading control.

**Fig 5:**
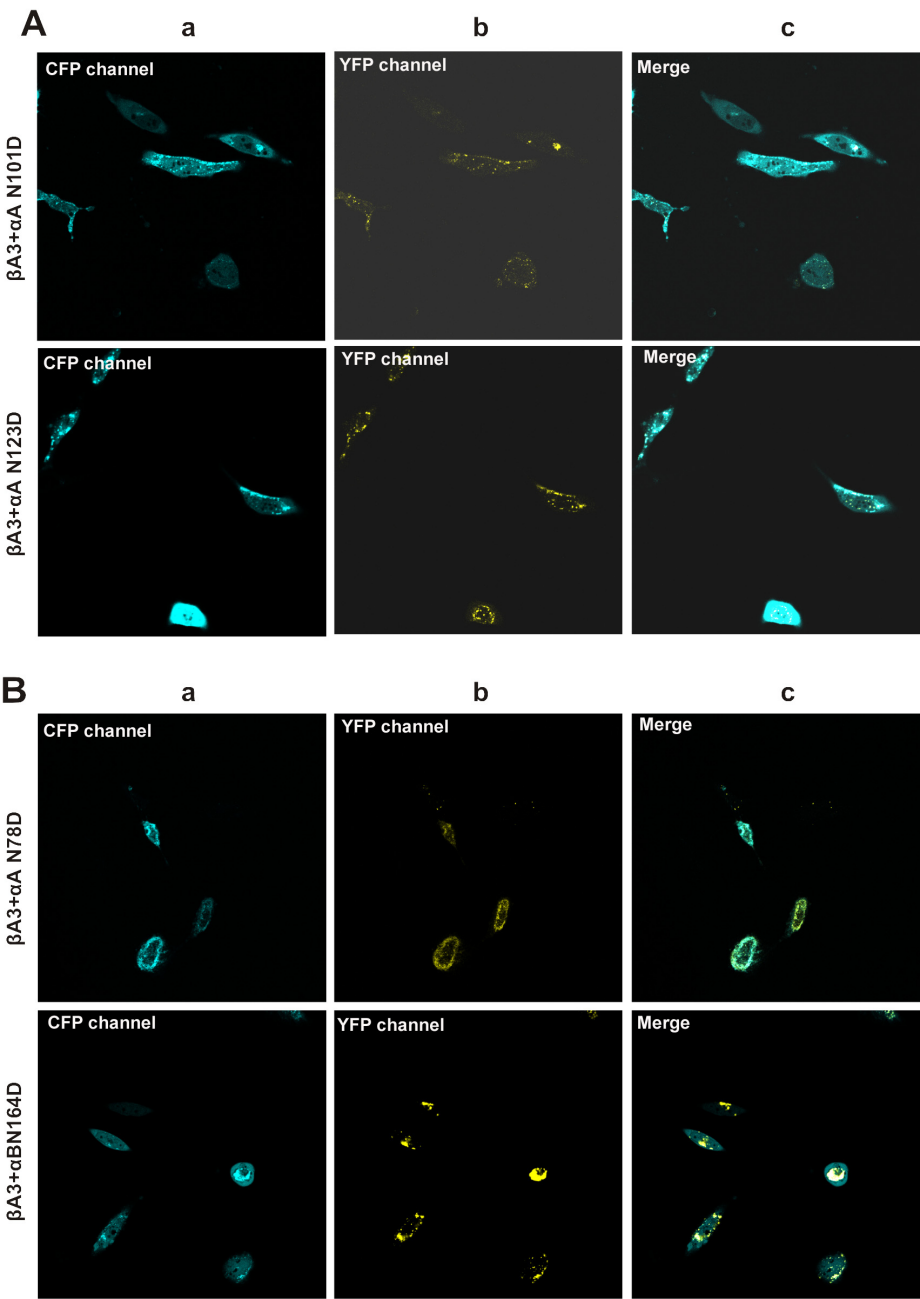
Confocal images of expression of deamidated mutants of αA- and αB-crystallins with βA3- crystallin in HeLa cells. Confocal microscopic images of HeLa cells co-transfected with: **A.** CFP βA3-crystallin-YFP αA N101D crystallin and YFP αA N123D crystallin **B.** CFP βA3-crystallin-YFP αB N78D crystallin and YFP αB N146D crystallin. Note the co-expression of CFP- and YFP-tagged crystallins. Panel a: CFP channel image of cells co-transfected with pairs of CFP- and YFP-fusion crystallins. Panel b: YFP channel image of cells co-transfected with pairs of CFP- and YFP-fusion crystallins. Panel c: Merged images for CFP and YFP channels of cells co-transfected with pairs of CFP- and YFP- fusion crystallin.

CFP βA3-crystallin was expressed in both cytoplasm and in the nucleus (Fig 4A, top panel in a), and no bleed-through was observed in the YFP channel (Fig 4A, panel b). αA- and αB-crystallins were expressed in cytoplasm (Fig 4A, panel b). The CFP βA3- and YFP αA-/YFP αB-crystallins were expressed together (Fig 4A, panel c) and were selected as a region of interest for FRET analysis. Western blot analysis showed that βA3-crystallin was expressed as ~51 kDa protein (Fig 4B lane 5 in a and b). αA- and αB-crystallins were expressed as ~46 kDa protein (Fig 4B lane 3 and 4 in a and b). As shown in Fig 4B, degraded βA3- (lane 5), αA-, and αB-crystallins (lane 3 and 4 in a and b) were also observed during western blot analysis. A significant decrease in degraded product of βA3-crystallin was observed in the presence of αA- and αB- crystallins (Fig 4B lanes 1 and 2). The deamidated mutants of αA- and αB-crystallins, YFP αA N101D, YFP αA N123D, YFP αB N78D, and YFP αB N146D were also expressed in the cytoplasm (Fig 5A and 5B, panel b).

**Fig 6:**
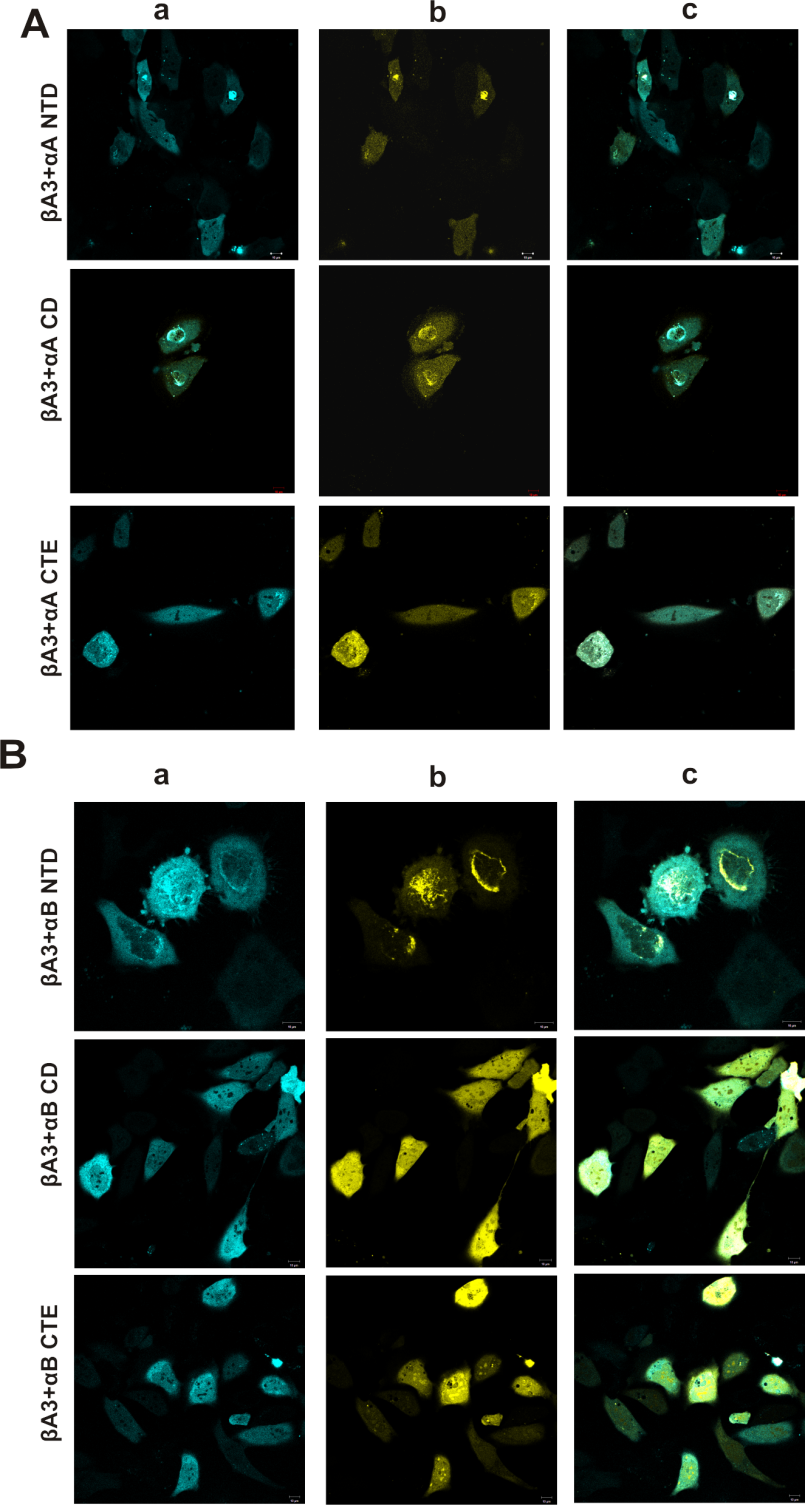
Confocal images of expression of domains of αA- and αB-crystallins with βA3-crystallin in HeLa cells. Confocal microscopic images of HeLa cells co-transfected with: **A.** CFP βA3-crystallin -YFP αA NTD, YFP αA CD and YFP αA CTE crystallin. **B.** CFP βA3-crystallin-YFP αB NTD, YFP αB CD and YFP αB CTE crystalline. Note the co-expression of CFP- and YFP-tagged crystallins.Panel a: CFP channel image of cells co-transfected with pairs of CFP- and YFP-fusion crystallins. Panel b: YFP channel image of cells co-transfected with pairs of CFP- and YFP-fusion crystallins. Panel c: Merged images for CFP- and YFP-channels of cells co-transfected with pairs of CFP- and YFP-fusion crystallins.

Among αA-domain mutants, YFP αA NTD was expressed as a cytoplasmic protein, whereas YFP αA CD and YFP αA CTE were expressed in both cytoplasm and the nucleus (Fig 6A, panel b). Among αB domain mutants, YFP αB NTD was expressed mostly around the nucleus and in the cytoplasm, whereas YFP αB CD and YFP αB CTE were expressed in both cytoplasm and the nucleus (Fig 6B, panel b).

#### FLIM-FRET analysis

**Fig 7:**
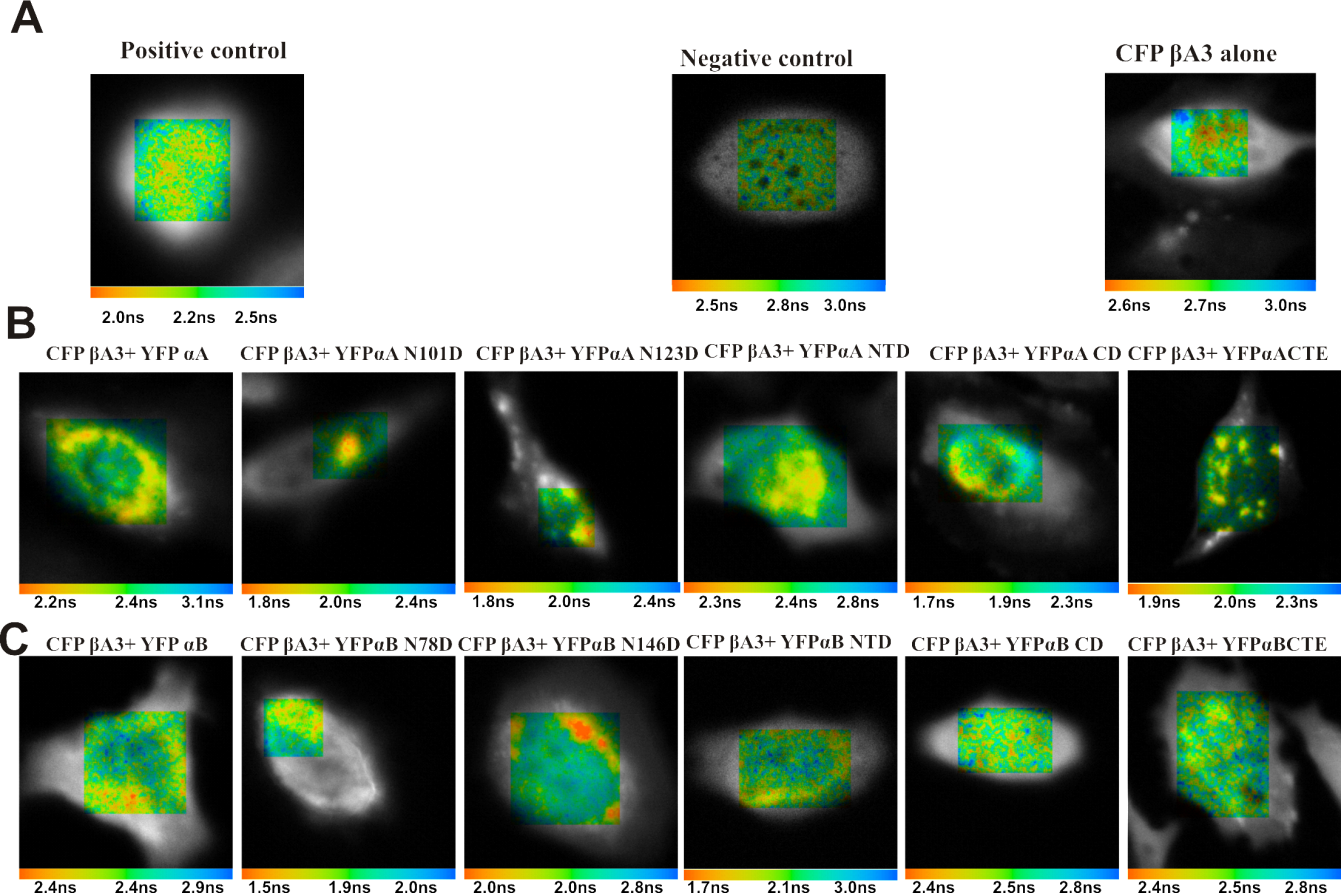
FLIM FRET images of HeLa cells showing life-time of the CFP βA3- crystallin in the presence of acceptor proteins (WT αA- and WT αB-crystallins and their deamidated and domain mutants). FRET measurement by fluorescence life time imaging microscopy of the HeLa cells transfected with **A.** Positive (YFP-CFP fusion), negative (CFP and YFP co-transfected) controls and CFP βA3-crystallin **B.** Co-transfected with CFP βA3-crystallin and YFP WT αA-crystallin and its mutants **C.** Co-transfected with CFP βA3-crystallin and YFP WT αB-crystallin and its mutants. The images were taken with Becker and Hickl FLIM system attached with the confocal miroscope. The life-time images are shown in pseudocolours (nanoseconds). The apparent mean life-time™ for each image is shown in the center at the bottom of each image.

Life-time of the CFP βA3-crystallin was determined in the presence and absence of acceptors (YFP αA-/YFP αB-crystallins and their mutants). Fig 7A demonstrates the life-time images of HeLa cells expressing positive control (CFP and YFP fusion) and negative controls [(CFP and YFP co-expressed)] and (CFP βA3-crystallin and YFP co-expressed)]. The positive control showed a decrease in the mean life-time of the CFP to 2.2 ±0.1 ns from 2.8±0.2 ns, which indicated that the energy was transferred from the CFP to the YFP. The life-time of the CFP βA3-crystallin was 2.8±0.2 ns, which did not change in the presence of YFP (Fig 7A). The mean life-time of the CFP was also 2.8±0.2 ns when co-expressed with the YFP (Fig 7A). These results showed that the life-time of the donor was 2.8±0.2 ns, which was used in the FRET efficiency calculations.

**Fig 8:**
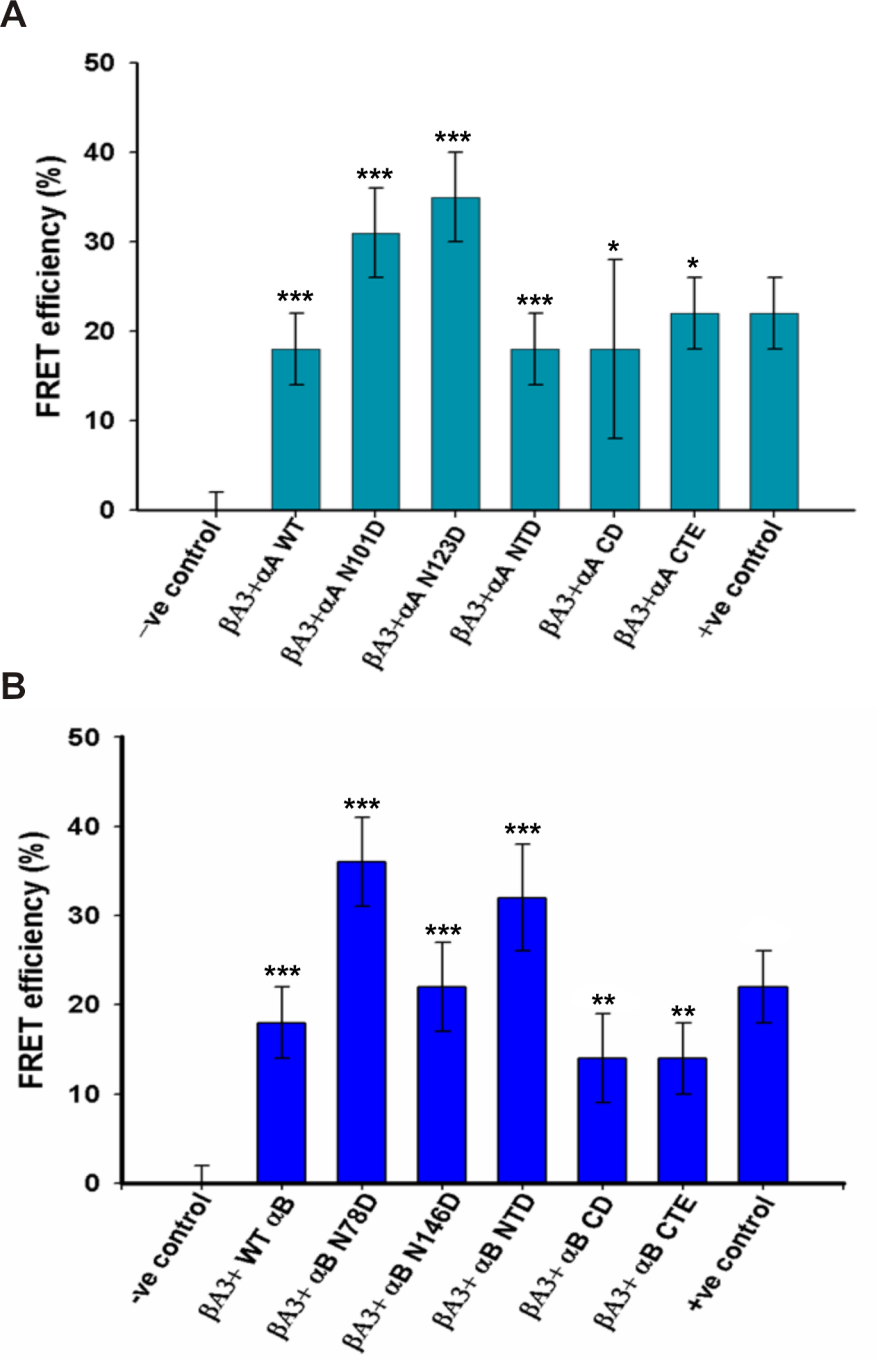
FLIM FRET efficiency of: (A) αA-crystallin (B) WT αB-crystallin, their deamidated and domain mutants. FRET efficiency was calculated from the decrease in life-lime (presented in Fig 8) in 5 to 6 cells. Each experiment was done in triplicate and average FRET efficiencies with standard deviation are presented. For FRET efficiency life-time of donor (T_CFP_) was 2.8 ns, which was the life-time of CFP βA3-crystallin in negative controls. The results are presented as mean ± standard deviation (SD). The FRET efficiency was significant at the level ***p≤ 0.0001 for WT αA, WT αB and their deamidated mutants, **p≤0.01 for WT αB, CD and CTE and *p≤0.05 for αA CD and CTE domains.

When the CFP βA3-crystallin was co-expressed with the WT YFP αA-crystallin, the mean life-time of the CFP βA3-crystallin was reduced to 2.4±0.1 ns from 2.8±0.2 ns, which suggested that the energy was transferred to the YFP WT αA-crystallin (Fig 7B). Similarly, in the presence of the WT YFP αB-crystallin, the CFP βA3-crystallin transferred energy, and the life-time was decreased to 2.4±0.2 ns from 2.8±0.2 ns (Fig 7C). The positive control showed 22±4% FRET efficiency. The CFP WT βA3-crystallin transferred an almost equal level of energy to the YFP WT αA-crystallin and the YFP WT αB-crystallin with a FRET efficiency of 18±4% for both (Fig 8A and 8B). The results suggested that the YFP WT αA- and YFP WT αB-crystallins were almost at an equal distance from the CFP WT βA3-crystallin, and, therefore, similar level of energy was transferred.

The life-time of the CFP WT βA3-crystallin was observed to be 2.0±0.2 ns and 1.8±0.2 ns in the presence of YFP αA N101D and YFP αA N123D, respectively (Fig 7B). YFP αA N101D and YFP αA N123D showed 32±4% and 36±4% FRET efficiency with the CFP βA3-crystallin, which was higher compared to the YFP WT αA-crystallin (Fig 8A) (p<0.05). An increase in the energy transfer of CFP βA3-crystallin to the deamidated αA-crystallin (YFP αA N101D and YFP αA N123D) compared to the WT YFP αA-crystallin was observed.

The CFP βA3-crystallin showed a life-time of 1.6±0.3 ns and 2.2±0.3 ns, respectively (Fig 7C) in the presence of the YFP αB N78D and YFP αB N146D crystallins. YFP αB N78D showed a higher FRET efficiency of 36±5% relative to the 18±4% efficiency of WT YFP αB-crystallin (p<0.001). However, YFP αB N146D showed 22±4% efficiency, which was slightly higher than WT YFP αB crystallin (Fig 8B) but the difference was not statistically significant. This suggested that more energy was transferred from the CFP βA3-crystallin to the YFP αB N78D mutant compared to the level of energy transferred to the YFP αB N146D and YFP WT αB-crystallins.

In summary, the WT YFP αA- and WT YFP αB-crystallins showed strong interaction to the CFP βA3-crystallin. Further, the deamidation of αA- and αB-crystallins increased the interaction efficiency with the CFP βA3-crystallin.

#### Domains of αA- and αB-crystallins are involved in interaction with βA3-crystallin

The CFP βA3-crystallin showed a life-time of 2.4±0.2 ns and 2.3±0.5 ns in the presence of YFP αA NTD and YFP αA CD, respectively, however, the life-time was reduced to 2.0±0.3 ns in the presence of YFP αA CTE (Fig 7B). YFP αA NTD and YFP αA CD showed identical FRET efficiency (18±4% and 18±9%, respectively), which was similar to the YFP WT αA-crystallin. However, YFP αA CTE showed a higher FRET efficiency of 22±5% relative to YFP WT αA-, YFP αA NTD, and YFP αA CD (Fig 8A) but the difference was statistically significant (p>0.05).

Among the domain mutants of the αB-crystallin, the life-time of the CFP βA3-crystallin was reduced to 2.1±0.3 ns from 2.8±0.2 ns in the presence of the YFP αB NTD. However, in the presence of YFP αB CD and YFP αB CTE, the life-time was 2.5±0.2 ns (Fig 7C). The life-time of the CFP βA3-crystallin was 2.4±0.2 ns in the presence of the WT YFP αB-crystallin, and the FRET efficiency of the YFP αB NTD was higher (32±6%) compared to the 14±4% of the YFP αB CD and YFP αB CTE and 18±4% of YFP WT αB-crystallins (Fig 8B) (p<0.05). The higher energy transfer to the αB NTD suggested that this region of αB might be involved in the interaction with βA3-crystallin.

## Discussion

Lens transparency is maintained by the solubility and interactions among crystallins. The interaction among crystallins is delicate and can be perturbed by PTMs or stress [4,5]. These perturbed interactions result in non-uniform changes in lens protein density and refractive index and lead to a subsequent increase in light scattering. Specific genetic mutations in crystallins, reported during cataract development, also affect the protein-protein interactions [6–10,49].

The covalent changes in lens crystallins disturb the non-covalent and weak interactions among them and may alter association among crystallins, which eventually leads to the development of lens opacity. The increased interaction was observed between covalently modified γB-crystallin and α-crystallin, while interaction of α-γ-crystallins, and α-β-crystallins was decreased during aging [5,50,51]. In our present study, αA- and αB-crystallins showed strong interaction with βA3-crystallin. WT αB-crystallin showed relatively higher binding with βA3-crystallin (Fig 3) than WT αA-crystallin (Fig 2). This is in confirmation with an earlier report when HMW β-crystallin, isolated from human lenses, was used in an interaction study [51]. However, when the interaction was studied under physiological condition using the FLIM-FRET method, both, αA- and αB-crystallin showed almost equal interaction with βA3-crystallin. Further, FRET results were consistent with our earlier findings in which βA3-crystallin (βA3 fused to GFP) interaction with WT αA- and WT αB-crystallins (fused to pDS red) was examined using a photobleaching FRET method [46]. In the previous and present studies, FRET efficiencies were almost equal even though FRET calculation methods by the two techniques were different. In the present study, FRET was calculated by measuring a decrease in donor life-time, whereas in the previous study [46], FRET was calculated by an increase in the intensity of the acceptor after photobleaching.

Deamidation in crystallins has been shown to cause structural changes, which have profound effects on protein-protein interactions [5,23,25,26]. Therefore, in this study, we examined the effects of deamidation of αA- and αB-crystallins on their interaction with βA3-crystallin in vitro and in vivo. The binding propensities of deamidated αA-crystallins were lower than WT αA-crystallin for βA3-crystallin (Fig 2). This result was expected since our earlier study showed that deamidation in αA-crystallin decreased the tryptophan spectra intensity, surface hydrophobicity, and formed more compact, but higher molecular weight oligomers, suggesting their altered conformation and assembly [27]. Therefore, we suggest that steric hindrance caused by the increased oligomer sizes is likely to be responsible for the lower binding of deamidated αA-crystallin relative to WT αA-crystallin. Similarly, relative to the WT αA-crystallin, the increased oligomer size and altered structures of the deamidated αA-crystallin mutants could be responsible for their closer proximity to βA3-crystallin, and for the relatively higher energy transfer to them compared to smaller size oligomers of WT αA-crystallin.

Deamidation of αB-crystallins has been shown to alter the secondary and tertiary structures, which results in the partitioning of deamidated αB-crystallin into water-insoluble fractions and formation of aggregates with other crystallins in aging and cataractous lenses [29,49]. In SPR analysis the αB N78D mutant showed a higher binding propensity to βA3-crystallin compared to WT αB- and the αB N146D mutant (Fig 3). Similar results were also observed during FLIM-FRET analysis. This result was surprising since our past study showed that αB N78D did not have any significant changes in structural and functional properties compared to WT αB-crystallin [29]. Therefore, the reason for the increased association of αB N78D to βA3-crystallin needs to be further investigated. In contrast, deamidation of αB-crystallin at position 146 has been shown to increase the surface hydrophobicity and an increase the oligomeric size relative to WT αB-crystallin [29]. This could be one of the reasons for comparatively lower binding affinity (Fig 3) and increased FRET efficiency of the αB N146D crystallin. Reports have shown that deamidated mutants of αA- and αB-crystallins (isolated from lenses) increased their surface hydrophobicity with higher molecular weight aggregates [20,52,53]. Therefore, we speculate that under physiological conditions, an increased surface hydrophobicity might have exposed the surface binding sites of the deamidated mutants of αA- and αB-crystallins, and, therefore they showed a stronger association with the βA3-crystallin. However, the discrepancies in binding affinity in our in-vivo and in-vitro studies need further investigation.

**Fig 9:**
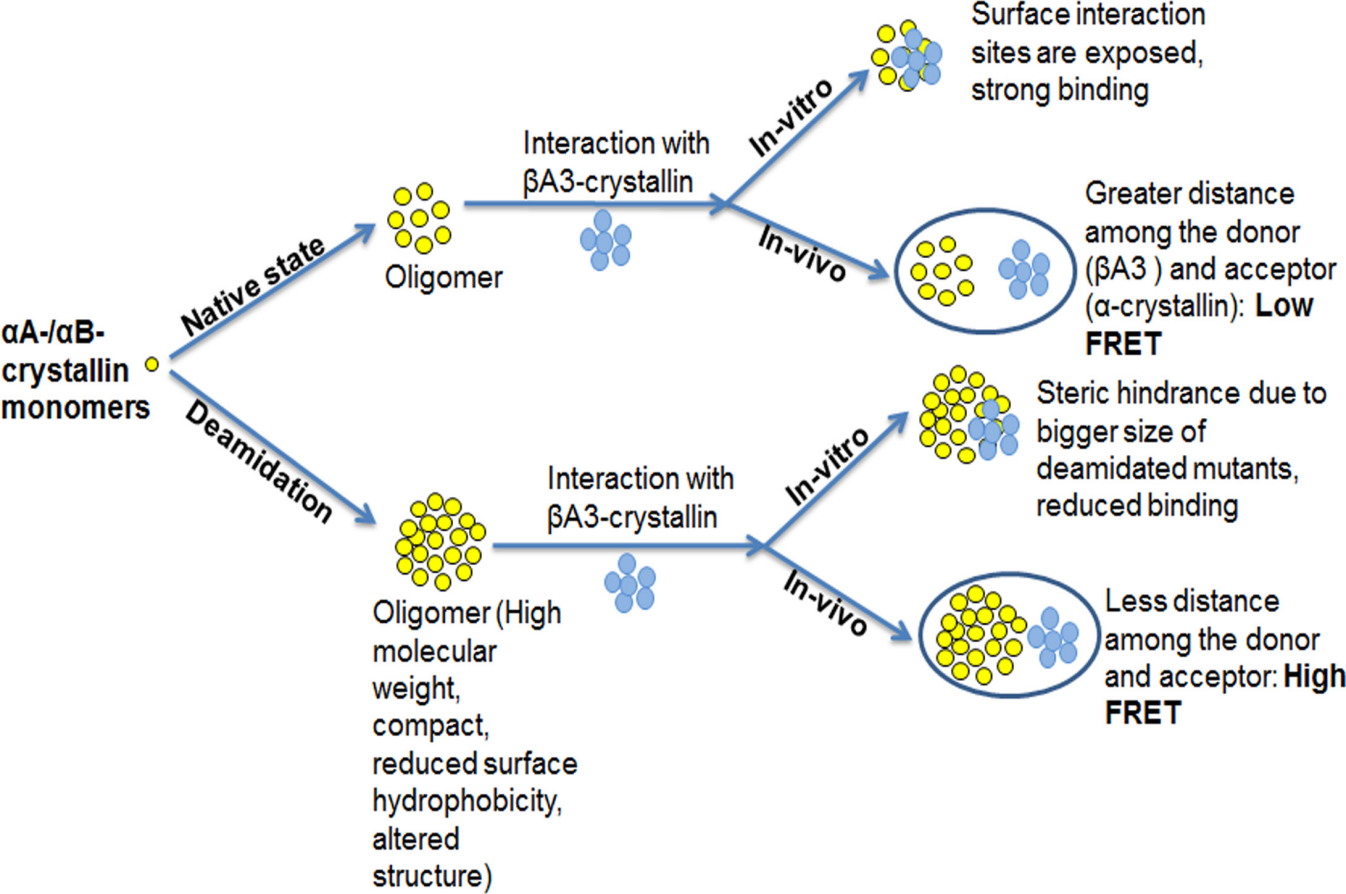
A diagrammatic representation of the in vitro and in vivo interaction of WT α-crystallin and their deamidated mutants with βA3-crystallin.

Based on these data and previous studies, we hypothesized that increased oligomer size of deamidated mutants may play an important role in interaction with βA3-crystallin as shown schematically (Fig 9). During in vitro interaction studies of deamidated mutants of α-crystallin, steric hindrance caused by their larger oligomers size and reduced surface hydrophobicity might result in loose binding with βA3-crystallin. However, under in vivo conditions, these larger oligomers are in close proximity with the βA3-crystallin in cells, which may have resulted in increased interaction of deamidated mutants of α-crystallin with βA3-crystallin.

With respect to the interactions of the NTD, CD and CTE domains of αA and αB-crystallin with βA3-crystallin, the FLIM-FRET data showed that αA CTE had a higher binding efficiency to βA3-crystallin compared to the binding efficiency of WT αA-crystallin to βA3-crystallin, whereas αA NTD and αA CD had almost equal FRET efficiency (Fig 8A). A previous study has shown that αA NTD and αA CD did not show any significant structural changes and retained their chaperone activity [47], therefore, the binding strength of both the domains to the βA3-crystallin did not vary. However, the CTE domain of αA-crystallin has been shown to be involved in oligomeric assembly [54,55], and it might have formed an oligomeric complex with βA3-crystallin that resulted in the higher FRET efficiency. Among the three domains of αB crystallin, αB NTD showed higher FRET efficiency compared to αB CD, αB CTE, and WT αB-crystallins. The NTD domain of αB crystallin was identified as an interacting region with the substrate, and it is more flexible and solvent accessible with higher chaperone activity [47,56]. Therefore, it may have shown higher affinity for βA3-crystallin in comparison to αB CD and αB CTE.

In summary, our study demonstrated that WT αA- and WT αB-crystallins as well as their deamidated mutants had strong interaction with βA3-crystallin. However, under in vivo conditions on deamidation in αA-crystallin at positions Asn 101 and Asn 123 and in αB-crystallin at positions Asn 78 and Asn 146 relatively increased the interaction with βA3-crystallin. Further, the results showed that all the three domains (NTD, CD, and CTE-) of WT αA- and αB-crystallins were involved in the interaction with βA3-crystallin, but αA CTE and αB NTD showed greater affinities for βA3-crystallin. The residues of α-crystallins (WT and deamidated mutants) involved in the interaction with βA3-crystallin will be investigated in the future studies.

## Supporting Information

S1 Fig Binding of WT αA-crystallin and deamidated mutants (αA N101D and αA N123D) with βA3-crystallin. Sensograms with the fitting curves (black lines) represent the association and dissociation at 5, 10, 15, 20 and 25 μM of analytes: **A.** WT αA- **B.** αA N101D and **C.** αA N123D mutants with the βA3-crystallin, respectively.
(TIF)

S2 Fig Binding of WT αA-crystallin and deamidated mutants (αB N78D and αB N146D) with βA3-crystallin. Sensograms with the fitting curves (black line) represented the association and dissociation at 5, 10, 15, 20 and 25 μM of analytes: **A.** WT αB-crystallin **B.** αB N78D C. αB N146D mutants with the βA3-crystallin.
(TIF)
