# Supplementary figures and images for: Interaction of βA3-Crystallin with Deamidated Mutants of αA- and αB-Crystallins
Source: PLoS One. 2015 Dec 11;10(12):e0144621. doi: 10.1371/journal.pone.0144621 (PMC4691197; doi:10.1371/journal.pone.0144621)

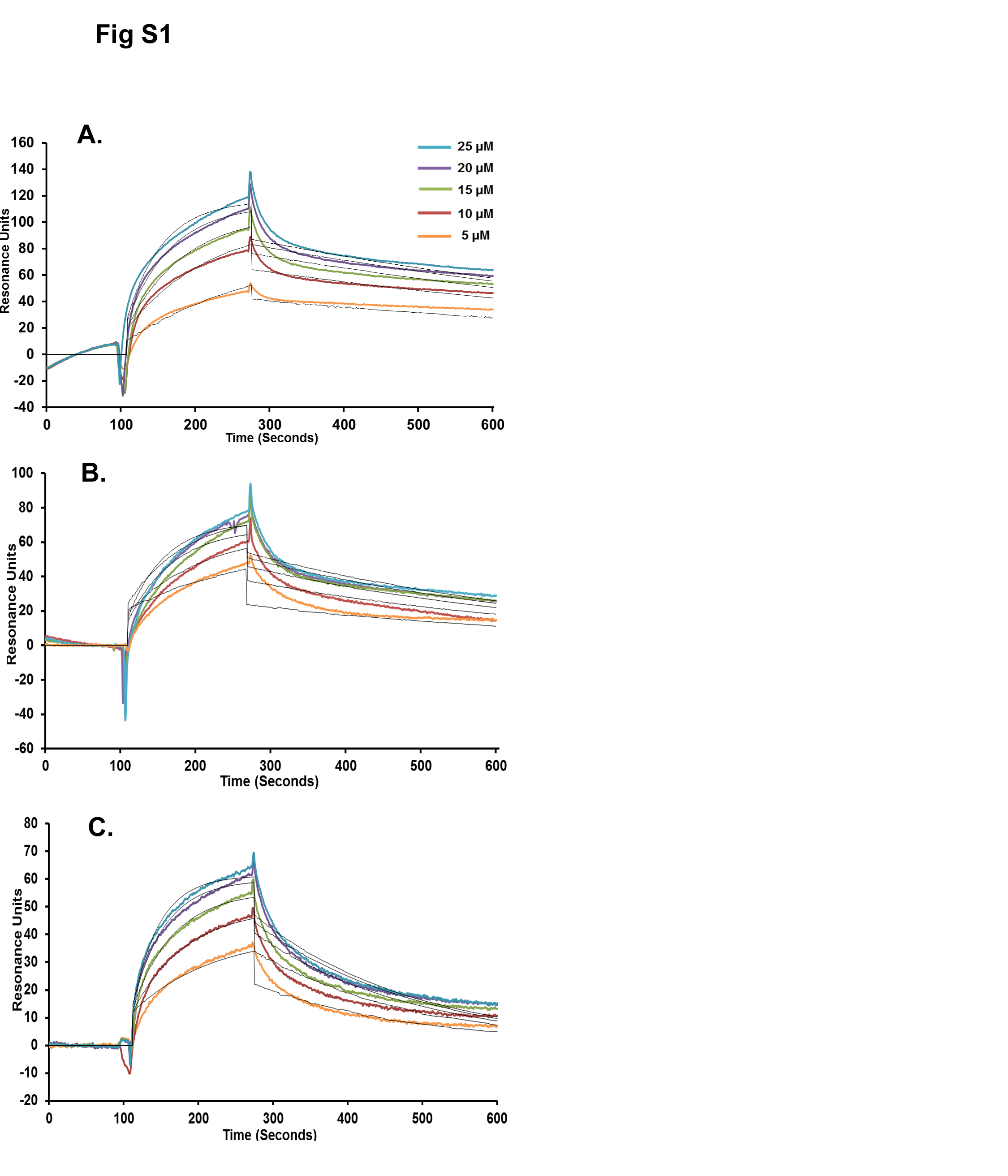

Supplement: S1 Fig — Sensograms with the fitting curves (black lines) represent the association and dissociation at 5, 10, 15, 20 and 25 μM of analytes: A. WT αA- B. αA N101D and C. αA N123D mutants with the βA3-crystallin, respectively. (TIF) [file pone.0144621.s001.tif]

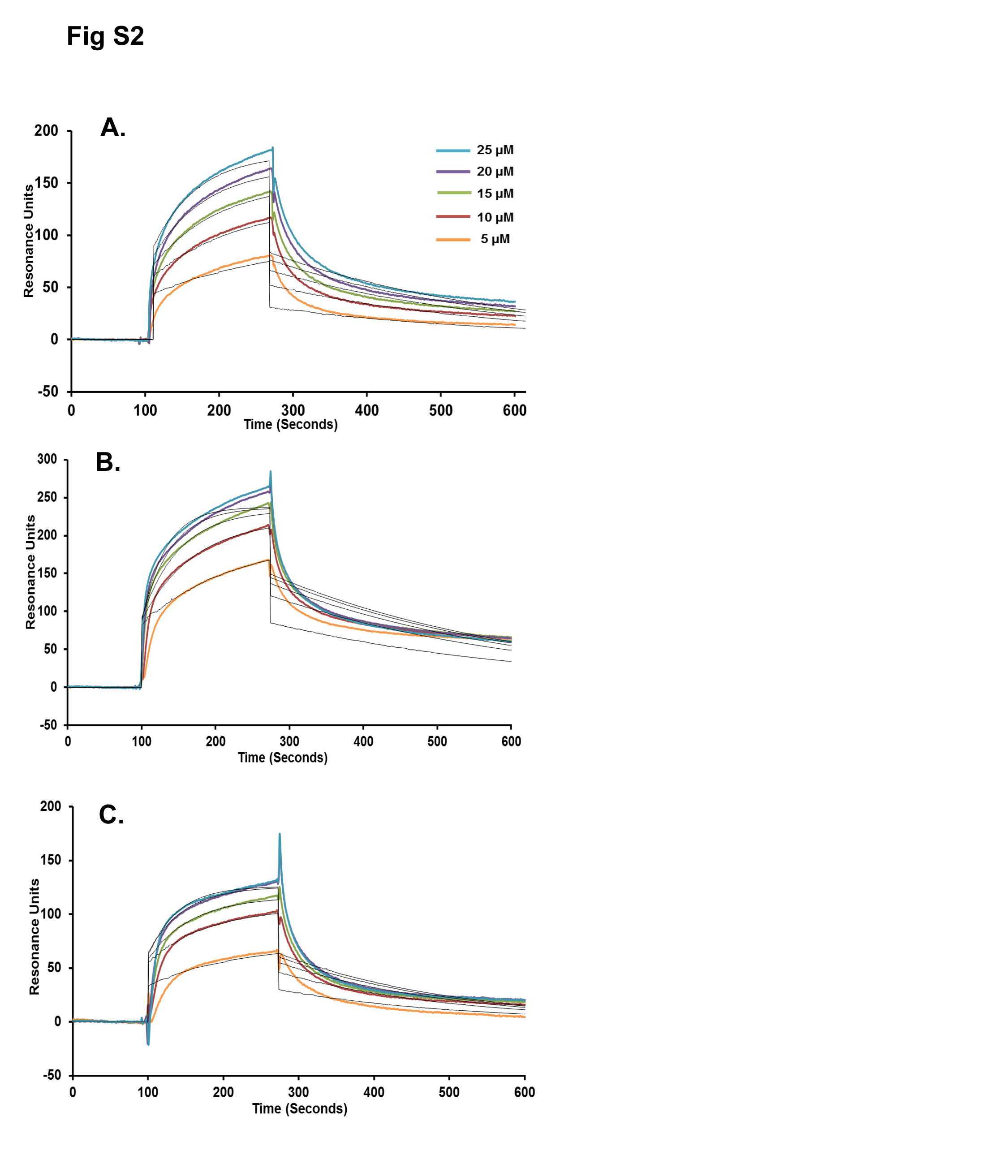

Supplement: S2 Fig — Sensograms with the fitting curves (black line) represented the association and dissociation at 5, 10, 15, 20 and 25 μM of analytes: A. WT αB-crystallin B. αB N78D C. αB N146D mutants with the βA3-crystallin. (TIF) [file pone.0144621.s002.tif]
